# Assessment of Quality of Life in Patients With Cardiovascular Disease Using the SF-36, MacNew, and EQ-5D-5L Questionnaires

**DOI:** 10.7759/cureus.17982

**Published:** 2021-09-14

**Authors:** Aikaterini Chatzinikolaou, Stergios Tzikas, Maria Lavdaniti

**Affiliations:** 1 Nursing Department, International Hellenic University, Thessaloniki, GRC; 2 Third Department of Cardiology, Ippokratio General Hospital, Aristotle University of Thessaloniki, Thessaloniki, GRC

**Keywords:** quality of life, cardiovascular disease, sf-36, eq-5d-5l, macnew questionnaire

## Abstract

Background

Cardiovascular disease (CVD) is responsible for 18 million annual deaths worldwide. CVD affects patients’ Quality of Life (QoL) mainly in physical, emotional and social dimension.

Aim

To assess the QoL of patients with CVD in Northern Greece, using three different instruments.

Methods

The study was conducted in one large hospital located in a major Greek city. A convenience sample of 80 patients participated. A questionnaire including Short Form-36 Health Survey (SF-36), EuroQoL 5-dimensions 5-levels (EQ-5D-5L), MacNew, demographic, and clinical characteristics was used to collect data.

Results

The mean age of the patients was 63.31±14.07. Analysis revealed statistically significant main effects of age on the physical limitations, emotional limitations, social functioning, and pain. Also, the analysis showed significant main effects of education on the MacNew Physical, MacNew Social, and the EQ-5D-5L index (p< 0.05). Participants who had graduated primary school had significantly lower quality of life scores than higher education graduates in the MacNew physical (p< 0.02). Furthermore, in the SF-36 pain subscale, the heart failure group had a significantly lower quality of life than the other heart diseases (p= 0.03)*.*

Conclusion

Quality of life is affected by factors such as age, type of heart disease, therapy, and comorbidities. Health care providers should be knowledgeable of the factors that affect the quality of life sectors (physical, emotional, and social life) of patients with CVD in order to meet their needs and have the most suitable treatment.

## Introduction

Cardiovascular disease (CVD) is the leading cause of morbidity and mortality [1] and is responsible for 18 million annual deaths worldwide [2]. CVD is the leading cause of death in Greece accounting for 48% of incidences [3]. Estimates suggest that by 2030, 44% of the population will have some type of CVD [4]. Patients with CVD experience numerous physical symptoms including fatigue, dyspnea, or chest pain which affect their physical, emotional, and social well-being with significant impairment in Quality of Life (QoL) [1,5].

QoL is defined by the World Health Organization (WHO) as “a broad-ranging concept affected in a complex way by the person’s physical health, psychological state, level of independence, social relationships and their relationship to salient features of their environment”[1]. QoL can be considered as one of the most important outcomes in healthcare, particularly among patients with CVDs. According to the WHO definition of health, QoL must be considered as a substantial health outcome in every disease management. The measurement of QoL provides an acceptable and valid method for assessing the impact of disease on patients’ function, activity, and well-being. Additionally, prior studies showed lower QoL among patients with CVD compared to the general population [6].

Additionally, comorbidities are another significant factor that is playing a leading role in the progression of the disease and determines the quality of life of each patient [7].

A cross-sectional study examined the impact of multimorbidity on QoL in 296 patients with CVD. QoL was assessed with the 36-item short-form (SF-36) health status survey. It was found that 69% of the patients suffered from coronary artery disease and had at least one additional disease such as diabetes mellitus or hypertension. The study also showed that the physical and mental component score of QoL was better in patients without any comorbidity. The physical component score in patients with higher educational levels was greater than those with low education levels [6].

Research using the SF-12 Short Form Healthy Survey showed that cardiovascular populations faced a majority of comorbidities. 5426 patients in the UK reported comorbidities such as hypertension, ischaemic heart disease, heart failure, and osteoarthritis. These findings were associated with poorer physical health and as a result with a poorer level of QoL [7].

In a more recent study, it was reported that the QoL of patients with coronary heart disease and angina was significantly lower compared to patients with myocardial infarction (MI). The study investigated 410 patients and used three different scales such as Heart QoL, SF-36, and HADS [8].

Previous studies have examined the factor of QoL in patients with CVD and found that the QoL of these patients was low. A study conducted in 2018, examined hopelessness and QoL of 200 patients with heart disease using the SF-36 QoL scale. It was found that elderly patients who suffered from CVD were affected negatively both physically and mentally, and as a result, they experienced decreased QoL [9].

Also, a study examined 53 heart failure patients using the health-related QoL (HRQOL) scale and showed that this group of patients experienced a variety of symptoms such as shortness of breath, lack of energy, difficulty in sleeping, and chest pain. All these explain why this group of patients mentions the lower quality of life [10].

Undoubtedly, there is growing interest in cardiovascular disease and the impact that it has on patient’s quality of life. In Greece, to the best of our knowledge, no research with these three scales of QoL has been conducted. The purpose of this study was to assess the QoL of patients with cardiovascular disease in Northern Greece using three different instruments. Specifically, we aimed to assess the following research questions:

Does QoL differ depending on demographic characteristics?

Does QoL differ depending on the type of cardiovascular disease?

Does QoL differ depending on the type of treatment?

Does QoL differ depending on the type of the existing comorbidities?

Do the three scales measure a patient’s QoL in the same way?

## Materials and methods

Study design and sample

This research study was conducted in the cardiology department of a large general public hospital in Northern Greece between April 2019 and September 2019. The sample was a convenience sample and consisted of 80 patients who suffered from cardiovascular disease. The inclusion criteria were age over 18 years, willingness to participate in the study, mental ability to complete the questionnaire, and the ability to speak and write in the Greek language. Out of 100 patients, 80 agreed to participate in the study (response rate: 80%). All eligible participants provided written, informed consent before completing a structured questionnaire.

Instruments

Short Form-36 Health Survey (SF-36)

The SF-36 health status survey is a 36-item, standardized quality of life assessment tool. It consists of 36 multiple choice questions that measure eight health constructs: physical functioning (10 items), physical role functioning (role physical)/role limitation due to physical problems (four items), bodily pain (two items), general perception of health (five items, energy and vitality (four items), social functioning (two items), emotional role functioning (role emotional) (three items) and mental health (five items). Scores are converted to a 0-100 scale, which allows us to measure the scales numerically. Higher values on the transformed 0-100 scale for each health domain indicate better health status. The lowest score indicates the lowest state of health and shows functional limitation, severe social and role disability, and distress. High scores indicate the absence of limitations and disability [11].

EuroQoL 5-dimensions 5-levels (EQ-5D-5L)

The EQ-5D-5L is a generic instrument for describing and valuing health. It is based on a descriptive system that defines health in terms of five dimensions: mobility, self-care, usual activities, pain/discomfort, and anxiety/depression. Each dimension has three response categories corresponding to no problems, some problems, and extreme problems. The instrument is designed for self-completion and respondents also rate their overall health on the day of the interview on a 0-100 hash-marked, vertical visual analogue scale (EQ-VAS) [12].

MacNew Questionnaire

The MacNew is a self-administered questionnaire designed to tap into a patient’s feelings about a range of issues and concerns identified by individuals with coronary heart disease (CHD), with norms available in patients with MI, angina, and heart failure. MacNew is designed to evaluate how physical, emotional, and social functioning, as well as daily activities, are affected by cardiovascular disease (CHD, ischemic heart disease [IHD], etc.). It includes 27 items in three domains (physical, emotional, and social) with a global score, has a two-week duration, and is scored from 1 (low HRQOL) to 7 (high HRQOL). The established minimal importance difference (MID) for the MacNew is 0.5 points on the 7-point scoring scale [13,14].

Data Analysis

Data were analysed using the SPSS-25 (IBM Corp, Armonk, USA). Qualitative variables were described as n (%) whereas continuous variables were presented as mean ± standard deviation (normal distributed) or as median (interquartile range) for the non-normally distributed data. The normality of distribution was assessed via the Lillifors (Kolmogorov-Smirnov) test whereas the homogeneity of variances was tested by using Levene’s test. The association between the SF-36, EQ-5D-5L, and MacNew scores was assessed by computing the Spearman rs correlation coefficient. Inferential statistics were used to assess the effects of the demographic and clinical characteristics of the cardiovascular patients on the summarized scores of the SF-36, EQ-5D-5L, and the MacNew. For the variables that met the assumptions of the parametric tests, differences in the means were assessed using one-way Analysis of Variance (ANOVA) or t-test for independent samples depending on the number of groups. After significant ANOVAs, post-hoc comparisons with a Sidak correction were performed. For the variables that did not meet the assumptions of the parametric tests, the non-parametric equivalents were used: the Kruskal-Wallis H test or Mann-Whitney U test depending on the number of groups. Significant H effects were followed by post-hoc multiple comparisons with a Dunn-Bonferonni correction. The significance level was set to p < 0.05.

## Results

Demographic-clinical characteristics and SF-36, EQ-5D-5L, and MacNew descriptives

The mean age of the 80 cardiovascular patients was 63.31±14.07 years old and most of them were males (65%). Most of the patients were diagnosed with either coronary disease (43.8%) or heart failure (31.3%). The majority of the patients received pharmacotherapy for treatment (63.7%) instead of surgery and the most common comorbid disease was hypertension (36.3%). The demographic and clinical characteristics of the sample are presented in Table 1 and Table 2.

**Table 1:** Demographic characteristics of the sample.

Characteristics	N	%
Gender		
male	52	65.0
female	28	35.0
Age		
< 55 years old	23	28.7
55-65 years old	19	23.8
66-72 years old	20	25.0
73 ≥ years old	18	22.5
Family status		
non-married (single, divorced, widowed)	20	25.0
married	60	75.0
Residence place		
Urban	62	77.5
semi-urban or rural	18	22.5
Living status		
Living alone	7	8.8
Living with others	73	91.3
Employment status		
unemployed	7	8.8
private sector employee	17	21.3
civil servant	4	5.0
domestic work	3	3.8
pensioner	48	60.0
other	1	1.3
Insurance status		
national insurance	64	80.0
private insurance	10	12.5
national and private insurance	3	3.8
non-insured	3	3.8
Educational status		
primary school	19	23.8
secondary school	41	51.2
higher education	20	25.0

**Table 2:** Clinical characteristics of the sample.

Characteristics	N	%
Heart Disease		
coronary disease	35	43.8
heart failure	25	31.3
Others (valvular heart disease, aortic aneurysm, atrial fibrillation, angina pectoris)	20	25.0
treatment		
pharmacotherapy	51	63.7
surgery	29	36.3
Comorbidities		
hypertension	29	36.3
diabetes	11	13.8
other comorbidities	11	13.8
no comorbidity	29	36.3

In Table 3, the mean scores of the SF-36, EQ-5D-5L, and MacNew are presented. In the SF-36, the highest score was in the pain subscale (75.25±32.48) whereas the lowest was on general health (45.69±21.75). The mean score of the MacNew was 4.41±1.07 whereas the index of the EQ-5D-5Lwas 0.54±0.32.

**Table 3:** Mean scores of SF-36, EQ-5D-5L, and MacNew. VAS: visual analogue scale

Questionnaire	Subscale	Mean	Std. Deviation
SF-36	Physical functioning	64.75	33.72
	Limitations due to physical health	64.38	42.24
	Limitations emotional problems	63.75	44.12
	Energy fatigue	48.44	24.89
	Emotional wellbeing	50.95	18.67
	Social functioning	64.22	34.55
	pain	75.25	32.48
	General health	45.69	21.75
MacNew	MacNew emotional	4.41	0.90
	MacNew physical	4.60	1.50
	MacNew social	4.43	1.41
	MacNew global	4.41	1.07
EQ-5D-5L	EQ-5D-5L index	0.54	0.32
	Health assessment VAS	67.45	19.42

Correlations between the SF-36, EQ-5D-5L, and the MacNew

To assess the relationship between the three different scales of quality of life, the Spearman correlation coefficient between the subscale of these measures was assessed. Table 4 shows the results for the correlations between the SF-36 and the EQ-5D-5L and between the MacNew and the EQ-5D-5L. All the SF-36 subscales were positively correlated with the EQ-5D-5L summary index (p < 0.05). The stronger correlation was between the SF-36 general health the EQ-5D-5L index (rs = 0.57, p < 0.01) whereas the weaker correlation was between the EQ-5D-5L index and theSF-36 Limitations due to physical health (rs = 0.25, p < 0.05). Moreover, most SF-36 subscales positively correlated with the health assessment VAS of the EQ-5D-5L (p < 0.05). However, the correlation between the SF-36 emotional wellbeing and the health assessment VAS was not significant (rs = 0.21). Moreover, there were relatively strong positive correlations between the MacNew subscales and the EQ-5D-5Lindex (p < 0.01). The strongest correlation was between the MacNew global score and the summary index (rs = 0.64, p < 0.01). Finally, the health assessment VAS was significantly and positively correlated with all MacNew subscales (p < 0.01). Table 5 shows the correlation between the SF-36 and the MacNew. All the correlations were positive (p < 0.01) and significant with the highest coefficient noticed between the SF-36 and the MacNew global (rs = 0.51, p < 0.01).

**Table 4:** Correlations between the SF-36 and the EQ-5D-5L and between the MacNew and the EQ-5D-5L. ** p < 0.01; * p < 0.05. VAS: visual analogue scale

	EQ-5D-5L index	Health assessment VAS
	rs	rs
SF-36 Physical functioning	0.466^**^	0.485^**^
SF-36 Limitations due to physical health	0.245^*^	0.327^**^
SF-36 Limitations emotional problems	0.291^**^	0.373^**^
SF-36 Energy fatigue	0.537^**^	0.509^**^
SF-36 Emotional wellbeing	0.570^**^	0.211
SF-36 Social functioning	0.433^**^	0.459^**^
SF-36 pain	0.382^**^	0.417^**^
SF-36 General health	0.572^**^	0.474^**^
MacNew emotional	0.570^**^	0.433^**^
MacNew physical	0.627^**^	0.486^**^
MacNew social	0.578^**^	0.443^**^
MacNew global	0.636^**^	0.470^**^

**Table 5:** Correlation between the SF-36 and the MacNew. ** p < 0.01; * p < 0.05.

	MacNew emotional	MacNew physical	MacNew social	MacNew global
	rs	rs	rs	rs
SF-36 Physical functioning	0.410^**^	0.560^**^	0.458^**^	0.507^**^
SF-36 Limitations due to physical health	0.406^**^	0.406^**^	0.404^**^	0.401^**^
SF-36 Limitations emotional problems	0.347^**^	0.380^**^	0.351^**^	0.367^**^
SF-36 Energy fatigue	0.550^**^	0.533^**^	0.530^**^	0.565^**^
SF-36 Emotional wellbeing	0.408^**^	0.238^*^	0.251^*^	0.324^**^
SF-36 Social functioning	0.376^**^	0.480^**^	0.403^**^	0.428^**^
SF-36 pain	0.435^**^	0.409^**^	0.349^**^	0.394^**^
SF-36 General health	0.509^**^	0.488^**^	0.498^**^	0.514^**^

Effects of demographic characteristics on QoL

Table 6 shows the median scores of the SF-36, MacNew, and the EQ-5D-5L of males and females. There were no statistical differences between females and males on any quality of life measure, except the pain subscale of the SF-36 in which males reported a higher quality of life score regarding pain than women (U = 546.000, p < 0.05).

**Table 6:** Effects of gender on quality of life. Data are presented as median (interquartile range), Mann-Whitney U test VAS: visual analogue scale

Quality of life	Males	Female	U	p
SF-36 Physical functioning	80 (60)	62.50 (62.50)	633,500	0.33
SF-36 Limitations due to physical health	68.75 (75)	75 (100)	617,500	0.23
SF-36 Limitations emotional problems	100 (100)	66.67 (91.67)	671,000	0.52
SF-36 Energy fatigue	50 (40)	45 (38.75)	683,500	0.65
SF-36 Emotional wellbeing	52 (31)	54 (28)	638,000	0.36
SF-36 Social functioning	75 (50)	62.50 (62.50)	663,500	0.50
SF-36 pain	100 (32.50)	68.75 (57.50)	546,000	0.04
SF-36 General health	42.50 (30)	47.50 (36.25)	659,000	0.49
MacNew emotional	4.61 (1.24)	4.33 (1.79)	576,500	0.13
MacNew physical	4.81 (2.19)	5.08 (2.86)	687,500	0.68
MacNew social	4.39 (1.55)	4.31 (3.11)	721,500	0.95
MacNew global	4.46 (1.40)	4.61 (2.05)	668,500	0.55
EQ-5D-5L index	0.64 (0.49)	0.56 (0.40)	691,500	0.71
Health assessment VAS	70 (25)	70 (34)	705,000	0.81

Table 7 shows the summarized scores of the QoL measures across the age categories. Analysis revealed statistically significant main effects of age on the physical limitations, emotional limitations, social functioning, and pain SF-36 subscales, the physical, social, and global MacNew subscales and both EQ-5D-5L indices (p < 0.05). Regarding the SF-36, post-hoc comparisons revealed that participants over 73 years old had significantly lower quality of life in physical limitation (p = 0.01) and emotional limitations than participants aged between 66-72 years old (p = 0.02) and participants younger than 55 years old had a lower score on pain than those aged 55-65 years old (p = 0.02). In the MacNew, post-hoc comparison revealed that in physical, participants over 73 had a lower score than those younger than 55 (p = 0.02) and those between 55-65 years old (p = 0.03) and in the social subscale, participants over 73 had a lower score than those younger than 55 (p = 0.02) and those between 55-65 years old (p = 0.02). In the global measure, participants over 73 years old had a lower score than those aged between 55-65 years old (p = 0.03). Finally, regarding the EQ-5D-5L, participants over 73 years had a significantly lower score in the summary index than those younger than 55 years old (p = 0.01), those aged 55-65 (p = 0.01), and aged 66-72 (p = 0.02) and in the VAS health assessment than those aged younger than 55 (p = 0.03). No other differences were noted. 

**Table 7:** Effects of age on quality of life. Data are presented as median (interquartile range) or mean±standard deviation. F: one-way ANOVA (Sidak post-hoc test), H: Kruskal-Wallis H test (Dunn-Bonferroni post-hoc test) ^a ^statistically different from <55 years old, ^b^ 55-65 years old, ^c^ 66-72, ^d^ ≥73 years old VAS: visual analogue scale

Quality of life	<55 years old	55-65 years old	66-72	73 ≥ old	H or F (3)	p
SF-36 Physical functioning	80 (60)	70 (40)	92.50 (53.75)	32.50 (87.50)	7.29	0.06
SF-36 Limitations due to physical health	75 (50)	100 (75.50)	100 (37.50)^d^	12.50 (100)^c^	9.98	0.02
SF-36 Limitations emotional problems	100 (66.67)	100 (66.67)	100 (0)^d^	0 (100)^c^	9.66	0.02
SF-36 Energy fatigue	50 (25)	50 (30)	47.50 (42.50)	30 (52.50)	2.44	0.49
SF-36 Emotional wellbeing	48 (28)	52 (32)	52 (35)	52 (24)	1.28	0.73
SF-36 Social functioning	75 (50)	75 (50)	75 (50)	37.50 (75)	8.54	0.04
SF-36 pain	60 (57.50)^b^	100 (0)^a^	100 (31.88)	73.75 (77.50)	11.26	0.01
SF-36 General health	45 (25)	45 (35)	40 (30)	40 (43.75)	2.05	0.56
MacNew emotional	4.57 (1.21)	5 (1.22)	4.75 (1.07)	4.25 (2.05)	5.76	0.12
MacNew physical	5.46 (2.23)^d^	5.23 (1.85)^d^	4.69 (1.85)	3.04 (3)^a,b^	10.76	0.01
MacNew social	5.08 (1.69)^d^	4.92 (2.23)^d^	4.31 (1.36)^d^	3.69 (2.89)^a,b^	11.41	0.01
MacNew global	4.89 (1.48)	4.85 (1.59)^d^	4.43 (1.04)	3.66 (2.20)^b^	9.68	0.02
EQ-5D-5L index	0.61±0.23^d^	0.62±0.29	0.6±0.24^d^	0.31±0.4^a,c^	F (3)= 5.01	0.03
Health assessment VAS	72.65±17.35^d^	68.95±21.51	70.75±13.21	55.56±21.82^a^	F (3) = 3.30	0.03

In Table 8, the summarized scores of the QoL measures across three educational levels are presented. Analysis revealed significant main effects of education on the MacNew physical, MacNew social, and the EQ-5D-5Lindex (p < 0.05). Participants who had graduated primary school had significantly lower QoL scores than higher education graduates in the MacNew physical (p < 0.02), the MacNew social (p = 0.04), and the EQ-5D-5L index (p = 0.03). No other differences between the education status groups were noticed.

**Table 8:** Main effect of education level on quality of life. Data are presented as median (interquartile range) or mean±standard deviation. F: one-way ANOVA (Sidak post-hoc test), H: Kruskal-Wallis H test (Dunn-Bonferroni post-hoc test) ^a^ statistically different from primary school, ^b^ secondary school, ^c^ higher education VAS: visual analogue scale

Quality of life	Primary school	Secondary school	Higher Education	H or F (2)	p
SF-36 Physical functioning	50 (80)	70 (60)	87.5 (38.75)	3.29	0.19
SF-36 Limitations due to physical health	75 (100)	100 (75)	75 (93.75)	0.48	0.78
SF-36 Limitations emotional problems	100 (100)	100 (83.33)	66.67 (91.67)	1.67	0.43
SF-36 Energy fatigue	40 (40)	50 (40)	47.50 (42.50)	1.26	0.53
SF-36 Emotional wellbeing	52 (24)	52 (30)	48 (37)	0.10	0.95
SF-36 Social functioning	62.50 (68.75)	62.50 (68.75)	75 (50)	0.62	0.73
SF-36 pain	100 (67.50)	100 (61.25)	100 (31.88)	2.20	0.33
SF-36 General health	40 (35)	45 (27.50)	45 (25)	2.16	0.34
MacNew emotional	4.50 (1.36)	4.57 (1.38)	4.79 (1.64)	0.61	0.74
MacNew physical	3.62 (2.69)^c^	4.77 (1.92)	5.62 (1.96)^a^	8.29	0.02
MacNew social	3.85 (2.62)^c^	4.46 (1.35)	5.27 (2.08)^a^	6.27	0.04
MacNew global	4.04 (1.93)	4.48 (1.17)	5.06 (1.53)	5.86	0.05
EQ-5D-5L index	0.38±0.25^c^	0.57±0.31	0.64±0.32^a^	3.89	0.03
Health assessment VAS	60.42±17.12^c^	69.71±18.77	69.5±19.42^a^	1.66	0.20

Table 9 presents the median scores of the three measures of QoL across the two different marital statuses. No statistical differences between married and non-married patients were reported.

**Table 9:** Marital status and quality of life U = Mann-Whitney U test, data are presented as median (interquartile range). VAS: visual analogue scale

Quality of life	Non married (single. Divorced. Widowed)	Married	U	p
SF-36 Physical functioning	60 (73.75)	77.50 (58.75)	535,000	0.46
SF-36 Limitations due to physical health	75 (100)	100 (75)	544,000	0.50
SF-36 Limitations emotional problems	100 (91.67)	100 (100)	558,500	0.61
SF-36 Energy fatigue	47.50 (38.75)	47.50 (40)	564,000	0.69
SF-36 Emotional wellbeing	48 (23)	52 (28)	511,500	0.32
SF-36 Social functioning	50 (81.25)	75 (50)	484,00	0.18
SF-36 pain	77.50 (65)	100 (38.13)	514,00	0.29
SF-36 General health	47.5 (33.75)	45 (30)	584,000	0.86
MacNew emotional	4.11 (2.08)	4.57 (0.86)	498,000	0.26
MacNew physical	4.04 (3.61)	4.92 (2.26)	533,00	0.46
MacNew social	4.20 (3.20)	4.62 (1.73)	516,000	0.35
MacNew global	4.35 (2.14)	4.63 (1.45)	503,500	0.28
EQ-5D-5L index	0.45 (0.59)	0.64 (0.38)	497,500	0.25
Health assessment VAS	75 (24)	70 (30)	555,500	0.62

Effects of clinical characteristics on quality of life

Table 10 shows the descriptives of QoL measures across the heart disease categories. In most variables, there were no statistically significant differences between groups. However, in the SF-36 pain subscale, the heart failure group had a significantly lower quality of life than the other heart diseases (p = 0.03).

**Table 10:** Effects of heart disease type on quality of life. Data are presented as median (interquartile range) or mean±standard deviation. F: one-way ANOVA (Sidak post-hoc test), H: Kruskal-Wallis H test (Dunn-Bonferroni post-hoc test), ^a^ statistically different from coronary disease, ^b^ heart failure, ^c^ other heart diseases.\ VAS: visual analogue scale

Quality of life	Coronary Disease	Heart Failure	Other heart diseases	H or F (2)	p
SF-36 Physical functioning	80 (55)	70 (50)	62.50 (62.50)	1.77	0.41
SF-36 Limitations due to physical health	100 (75)	75 (100)	100 (43.75)	1.57	0.46
SF-36 Limitations emotional problems	100 (100)	100 (66.67)	100 (100)	0.21	0.90
SF-36 Energy fatigue	55 (40)	40 (37.50)	50 (40)	1.80	0.41
SF-36 Emotional wellbeing	52 (28)	52 (24)	42 (35)	2.97	0.23
SF-36 Social functioning	75 (50)	50 (37.50)	81.25 (75)	4.04	0.13
SF-36 pain	100 (55)	67.50 (61.25)^c^	100 (15)^b^	7.36	0.03
SF-36 General health	50 (35)	45 (30)	45 (28.75)	3.36	0.19
MacNew emotional	4.59±0.91	4.27±0.89	4.29±0.87	F= 1.22	0.30
MacNew physical	4.77±1.62	4.5±1.47	4.42±1.32	F= 0.44	0.65
MacNew social	4.55±1.5	4.38±1.36	4.27±1.36	F= 0.26	0.77
MacNew global	4.56±1.18	4.33±1.05	4.27±0.88	F= 0.57	0.57
EQ-5D-5L index	0.64 (0.49)	0.64 (0.46)	0.52 (0.48)	F= 0.58	0.75
Health assessment VAS	70 (30)	70 (30)	65 (28)	F= 0.07	0.96

Table 11 presents the median scores of the three measures of QoL according to the type of treatment received. No statistical differences were found between pharmacotherapy and surgery treatment groups.

**Table 11:** Type of treatment and quality of life. U = Mann-Whitney U test, data are presented as median (interquartile range). VAS: visual analogue scale

Quality of life	Pharmacotherapy	Surgery	U	p
SF-36 Physical functioning	80 (55)	55 (72.50)	624,500	0.24
SF-36 Limitations due to physical health	100 (75)	75 (87.50)	689,000	0.59
SF-36 Limitations emotional problems	100 (66.67)	100 (100)	675,500	0.48
SF-36 Energy fatigue	45 (35)	50 (37.50)	632,500	0.28
SF-36 Emotional wellbeing	48 (28)	52 (30)	641,000	0.32
SF-36 Social functioning	62.50 (50)	75 (75)	736,000	0.97
SF-36 pain	100 (55)	100 (43.75)	725,000	0.87
SF-36 General health	45 (30)	45 (30)	718,500	0.83
MacNew emotional	4.57 (1.36)	4.57 (1)	640,000	0.32
MacNew physical	4.85 (2.62)	4.92 (2.04)	642,500	0.33
MacNew social	4.46 (2.77)	4.46 (1.31)	659,000	0.42
MacNew global	4.44 (1.67)	4.63 (1.24)	686,500	0.60
EQ-5D-5L index	0.58 (0.37)	0.64 (0.59)	721,000	0.85
Health assessment VAS	70 (30)	70 (30)	597,500	0.15

Table 12 presents the median scores of the three measures of QoL according to the comorbidities. No statistical differences between the comorbidities groups were reported.

**Table 12:** The effect of comorbidities on quality of life. Data are presented as median (interquartile range) or mean±standard deviation. F: one way ANOVA (Sidak post-hoc test), H: Kruskal-Wallis H test (Dunn-Bonferroni post-hoc test) VAS: visual analogue scale

Quality of life	Hypertension	Diabetes	Other comorbidities	No comorbidity	H or F (3)	p
SF-36 Physical functioning	75 (60)	55 (75)	80 (60)	70 (67.50)	0.29	0.96
SF-36 Limitations due to physical health	75 (75)	75 (100)	100 (100)	75 (87.50)	0.58	0.90
SF-36 Limitations emotional problems	100 (100)	66.67 (100)	100 (33.33)	66.67 (100)	1.47	0.69
SF-36 Energy fatigue	50 (35)	40 (50)	30 (35)	60 (37.50)	4.86	0.18
SF-36 Emotional wellbeing	52 (28)	64 (36)	40 (24)	48 (28)	4.97	0.17
SF-36 Social functioning	62.50 (75)	75 (50)	75 (75)	75 (50)	1.12	0.77
SF-36 pain	77.50 (61.25)	100 (40)	100 (55)	100 (38.75)	1.82	0.61
SF-36 General health	40 (27.50)	45 (35)	25 (50)	50 (22.50)	3.11	0.37
MacNew emotional	4.57 (1.38)	4.71 (2.43)	4.57 (1.15)	4.57 (1.21)	1.29	0.73
MacNew physical	4.85 (2.43)	4.23 (4.07)	5 (2.53)	4.92 (2.12)	0.89	0.83
MacNew social	4.31 (2.07)	3.69 (3.39)	4.92 (1.77)	4.77 (1.92)	1.56	0.67
MacNew global	4.41 (1.61)	4.30 (3.26)	4.44 (1.67)	4.74 (1.15)	0.54	0.91
EQ-5D-5L index	0.56±0.3	0.51±0.37	0.56±0.3	0.53±0.33	F= 0.11	0.95
Health assessment VAS	62.07±17.45	69.64±21.81	65.91±29.48	72.59±14.8	F=1.52	0.22

## Discussion

The objective of the study was to assess the QoL of Greek patients who suffered from cardiovascular diseases. The study contributes to the growing body of evidence regarding the correlation between QoL in patients with CVD and provides important information for Greek cardiology nurses because describing this phenomenon is a significant step toward appropriate information. In this study, we found significant main effects on physical limitations, emotional limitations, social functioning, and pain among CVD patients.

In this study, we found that participants who had graduated from primary school had significantly lower QoL scores than higher education graduates in the MacNew physical, MacNew social, and the EQ-5D-5L Index. This finding is consistent with another study, which showed that participants with lower educational levels had lower physical component scores of QoL compared with the participants with higher levels of education [6].

We also found that the age group of >73 reported lower QoL because of physical and emotional limitations that were reported compared to other age groups, such as 66-72 years old. This is an expected outcome that is also confirmed by the findings of other studies which showed that QoL is severely affected to the elderly population [9,15].

Furthermore, it was found that in the MacNew social subscale participants over 73 years old had a lower score than those younger than 55 and those between 55-65 years old. Lack of physical activity and social functioning constitutes an expected finding as the elderly often face problems like these according to the international literature [16].

We found that there was no significant difference in QoL between women and men. This finding is inconsistent with other studies which indicated that women tend to have worse QoL compared to men. Previous studies have indicated that women tend to have worse QoL than men [17,18].

Our study also revealed that males had a higher QoL score on the pain subscale of the SF-36, compared to women. Therefore, women faced problems with pain more often than men, which shows that gender affects pain. This is consistent with a previous study in which participants reported the problem of pain. To the best of our knowledge women have often reported pain as a symptom in cardiovascular disease as general discomfort, abdominal pain, arm pain, or chest pain [19]. Also, according to the literature, pain is one of the most commonly occurring symptoms in patients with CVD and it is reported in 90% of the patients [10].

According to our findings, the heart failure group reported a significantly lower quality of life than the other heart disease groups. This finding is confirmed by other studies which have also reported that heart failure patients experience multiple physical and psychological symptoms that can significantly affect their QoL. Heart failure patients face a wide variety of symptoms that can have a negative impact on their everyday life such as dyspnea, fatigue, pain, orthopnea, edema, loss of appetite, anxiety, and depression which are widely reported [14].

To conclude, a significant finding which concerns the scales is the strong correlation between the SF-36 and the EQ-5D-5L scale and between the SF-36 and the MacNew questionnaire. This answers the hypothesis on whether the three scales measure patients' QoL in the same way.

Limitations

This study has some limitations. It was conducted in one hospital located in a major Greek city and the sample was relatively small, so the results cannot be generalized to the entire Greek population. Another limitation was the small timeline of the study which was conducted in a six months period. However, the results provide valuable information for the issue at hand and illustrate the great need for further research in order to draw reliable conclusions. Despite these limitations, our study has one significant strength. To the best of our knowledge, this is the first population-based study to use the combination of these three scales - SF-36, EQ-5D-5L, and the MacNew questionnaire in order to investigate the quality of life of CVD patients in Greece, where the culture and lifestyle are significantly different from those in Western populations. It is obvious that if data collection was conducted nowadays, maybe the results would be different because of the COVID-19 pandemic and its impact on CVD patients and the cardiovascular system in general.

## Conclusions

Our study confirms that the QoL of patients with CVD in Greece is significantly affected. Both pain as well as limitations on physical, emotional, social functioning, and pain influence the quality of life of these patients. Comorbidities and the level of education play a significant role in the perception of the quality of life of the participants. As far as we know, there has not been any study conducted that examined QoL simultaneously using these three scales (SF-36, EQ-5D-5L, and MacNew) in Greece. There is a growing interest in CVD patients and the impact that it has on patients’ QoL. Nevertheless, further research is needed to evaluate the QoL of patients with CVD in the Greek population. The results of the present study should help healthcare professionals expand their knowledge in order to attempt to treat patients who suffer from CVD through a holistic approach and as a result to improve patients' quality of life.
